# Complement activation in patients with post-acute sequelae after SARS-CoV-2 infection

**DOI:** 10.3389/fimmu.2026.1779393

**Published:** 2026-05-13

**Authors:** Madlene Holmqvist, Dick J. Sjöström, Katherine Carlson, Birgitta Gullstrand, Anders A. Bengtsson, Robin Kahn, Tom E. Mollnes, Per Åkesson, Per H. Nilsson, Fredrik Kahn

**Affiliations:** 1 Division of Infection Medicine, Department of Clinical Sciences, Lund University, Lund, Sweden; 2 Department of Infectious Diseases, Skåne University Hospital, Lund, Sweden; 3 Department of Chemistry and Biomedical Sciences, Linnaeus University, Kalmar, Sweden; 4 Division of Rheumatology, Department of Clinical Sciences, Lund University, Lund, Sweden; 5 Division of Pediatrics, Department of Clinical Sciences Lund, Lund University, Lund, Sweden; 6 Wallenberg Centre for Molecular Medicine, Lund University, Lund, Sweden; 7 Institute of Immunology, Oslo University Hospital and University of Oslo, Oslo, Norway; 8 Research Laboratory, Nordland Hospital Trust, Bodø, Norway

**Keywords:** complement activation, COVID-19, long COVID, PACS, PASC, post COVID syndrome, SARS-CoV-2

## Abstract

**Introduction:**

Post-acute sequelae of SARS-CoV-2 (PASC) may develop after SARS-CoV-2 infection and cause a wide range of symptoms that can persist for years. Several pathophysiological mechanisms have been proposed, including dysregulation of the complement system.

**Methods:**

In this study, we analysed markers of complement activation in a cohort of patients with PASC, up to 33 months after the initial infection. We measured the complement activation markers C3bc, C3bBbP and TCC in 38 PASC patients with an initial mild COVID-19 infection, 10 PASC patients with an initial severe COVID-19 infection and 80 control subjects who had recovered completely after a COVID-19 infection.

**Results:**

Although the patients with an acute mild SARS-CoV-2 infection had a trend towards more severe PASC, we could not find any significant differences in complement activation markers between these patients and controls.

**Conclusion:**

We could not find convincing evidence of activation of the complement system in PASC patients.

## Introduction

Post-acute sequelae of SARS-CoV-2 (PASC) is a syndrome that quickly became recognized as a clinical entity during the COVID-19 pandemic. Also known as “long covid”, it occurs in up to one out of eight people with a SARS-CoV-2 infection, affecting an estimated 65 million individuals worldwide (1, 2). Although lacking an exact definition, PASC is often described as persistent or new symptoms that usually last for at least two months after an infection with SARS-CoV-2 and cannot be explained by an alternative diagnosis (3). The symptoms often include fatigue, dyspnoea and cognitive dysfunction, which generally have an impact on everyday activities (3). PASC does not only affect individuals with an initial severe SARS-CoV-2 infection; in fact, the majority of patients with a diagnosis of PASC had a mild acute SARS-CoV-2 infection (4, 5). Several pathophysiological mechanisms have been proposed, including immune dysregulation. T cell alterations, elevated levels of cytokines and autoantibodies are among proposed immune mediated mechanisms (6, 7). In addition, changes in activation or function of the complement system have been reported in samples from this patient group (8–12). In the present study, we evaluated and compared markers of complement activation in patients with PASC after mild or severe SARS-CoV-2 infection who were recruited from a specialized PASC clinic up to 33 months after infection.

## Material and methods

**Figure 1:**
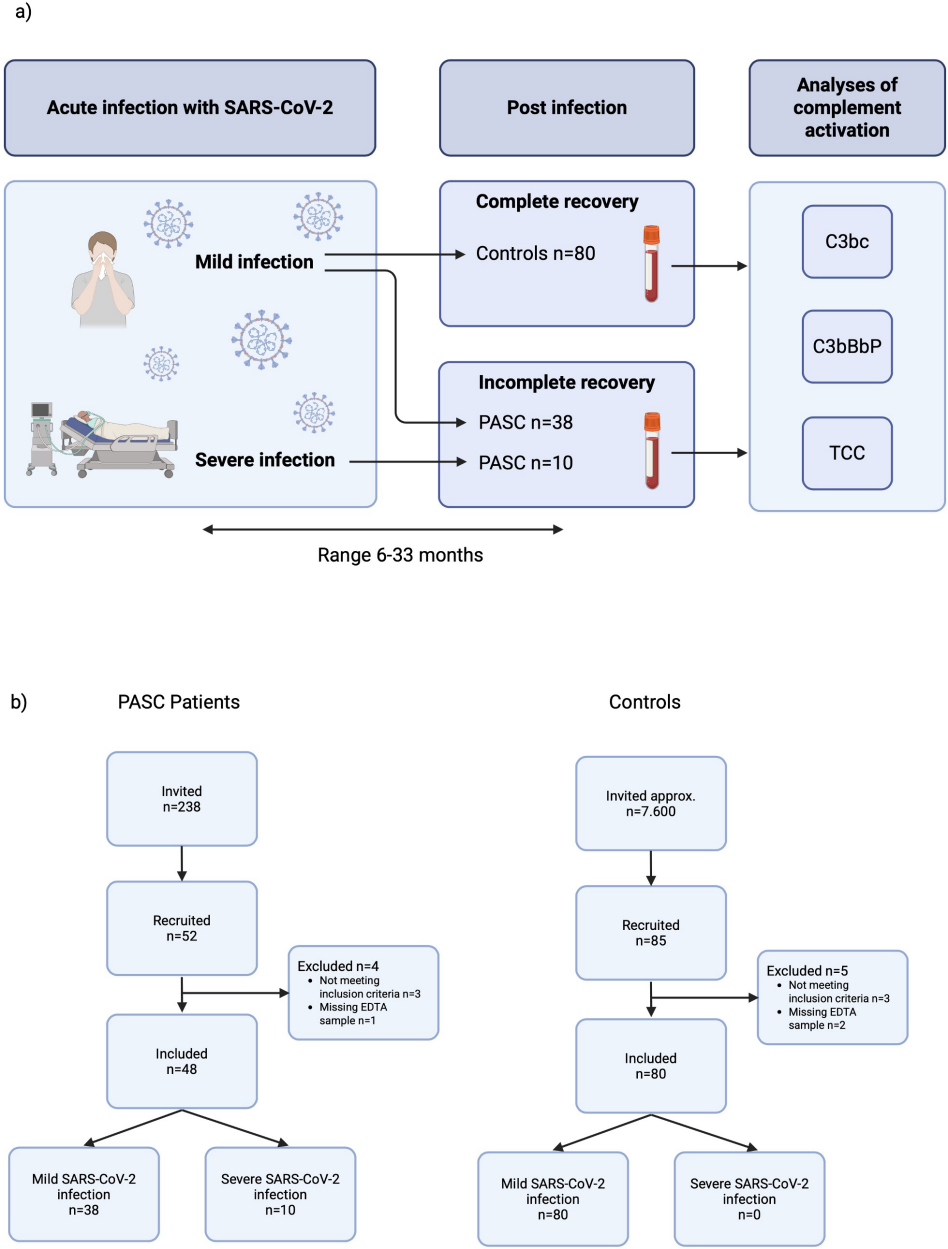
**(a)** Overview of study design. **(b)** Flow chart for inclusion of PASC patients and control subjects. Legend: PASC, Post-Acute Sequelae of SARS-CoV-2. Mild SARS-CoV-2 infection = not requiring oxygen therapy. Severe SARS-CoV-2 infection = requiring oxygen therapy. Figure created in BioRender. Holmqvist, (M) (2026) https://BioRender.com/9tfemyp.

An overview of the study design is shown in **Figure 1a**.

### Study populations

#### PASC patients

All patients aged ≥18 years who attended the PASC outpatient clinic at the Department of Infectious Diseases, Skåne University Hospital, a tertiary care centre serving 1.7 million inhabitants, were invited to participate. Additional inclusion criteria were a test confirming the infection with SARS-CoV-2 (PCR test, antigen test or serology, the latter not as a result of vaccination) and symptoms persisting for at least 12 weeks after the infection. Part of the cohort has previously been described (13). At inclusion, the patients underwent an interview about their associated symptoms, cognitive testing and blood sampling and they completed questionnaires related to quality of life and symptoms from specific organ systems. The patients were classified into two groups: (1) mild acute infection, defined as no requirement for oxygen therapy during the acute SARS-CoV-2 infection, and (2) severe acute infection, defined as requiring supplemental oxygen therapy during the acute infection, corresponding to WHO clinical progression scale 1–4 and 5–9, respectively (14). All patients were invited for follow-up visits after 6–12 months, when the same study protocol was repeated, including follow-up plasma sampling.

#### Control subjects

Control subjects aged ≥18 years were recruited from other study cohorts with a known history of SARS-CoV-2 infection (15). Control subjects had fully recovered, reported no persistent symptoms after SARS-CoV-2 infection, and underwent the same testing procedures as the PASC patients.

### Sampling and complement analyses

Blood samples were collected from patients and controls in 5 ml EDTA-tubes (BD) and centrifuged for ten minutes at 2000 x g to obtain plasma. Plasma samples were subsequently stored at -80 °C until further use. The complement activation products C3bc, C3bBbP and TCC were measured as markers of activation of the complement system. C3bBbP is a marker specific for activation of the alternative pathway. C3bc is a marker of C3-cleavage and terminal complement complex (TCC) is a marker for complement activation to its very end; both are common to activation of all pathways (classical, lectin and alternative pathways). The assays have been described previously in detail (16). The reference ranges for C3bc, C3bBbP and TCC are <9 CAU/mL, <24 CAU/mL and <0.7 CAU/mL, respectively (16).

### Ethical approval

The present study was approved by the Swedish ethical review authority, (Dnr 2021-03905). All patients and control subjects gave their informed consent to participate in the study. The authors assert that all procedures contributing to this work comply with the ethical standards of the relevant national and institutional committees on human experimentation and with the Helsinki Declaration of 1975, as revised in 2008.

### Statistics

Statistical analyses were performed using GraphPad Prism (version 10.3.1 for Mac, GraphPad Software, San Diego, CA), IBM SPSS Statistics (version 31.0.0.0) and R (version 4.4.2) with the packages readxl, MuMin, car (R Core Team (2023). *R: A Language and Environment for Statistical computing.* R Foundation for Statistical Computing, Vienna, Austria.). Medians, interquartile ranges (IQRs), minimum and maximum values were reported when appropriate. Differences between groups were analysed using the Mann–Whitney U test, Wilcoxon signed-rank test, and Fisher’s exact test, as appropriate. P-values less than 0.05 were deemed statistically significant. In the primary analysis, where only the first sample from each person was included, unadjusted p-values were employed. In the secondary analysis where both the first and the second sample was included, p-values were adjusted using the Holm-Bonferroni method to accommodate multiple comparisons. Participants with missing follow-up blood samples were excluded from the secondary analyses, and observations with missing demographic data were omitted from the relevant analyses. In the sensitivity analyses, multivariable linear regression models were used with log-transferred dependent variables.

## Results

### Patient characteristics

Forty-eight patients with PASC and 80 control subjects were included in this study. The flow chart for inclusion is displayed in **Figure 1b**. The patients with long-term symptoms were divided into two groups based on the severity of the acute infection: 1) mild (non-oxygen dependent) and 2) severe (oxygen-dependent). The reason for this grouping was the differences in clinical presentation and demographics (**Table 1**). The pathophysiology for long-term symptoms may also differ between these two groups. Ten patients had severe acute infection, whereas 38 patients had mild acute infection. Only one patient with mild SARS-CoV-2 infection was admitted to hospital during the acute infection. None of the control subjects were hospitalized during the acute infection. Time from infection with SARS-CoV-2 to first plasma sampling was a median of 16 months (range 6–26 months) for PASC patients with a severe infection, 15 months (range 9–24 months) for PASC patients with a mild infection, and 10 months (range 5–27 months) for the control group. A second plasma sample was collected from 20 (53%) of the patients with a mild infection and in 6 (60%) of those with a severe infection after a median of 8 months (range 7–10 months) from the first sample.

**Table 1:** Demographics of the study population.

Variable	PASC (mild COVID-19) n=38	PASC (severe COVID-19) n=10	Controls n=80
Age, median (min-max)	46 (25-63)	55 (41-78)	54 (28-69)
Female sex, n (%)	27 (71%)	6 (60%)	59 (74%)
BMI, median (min-max)	26.8 (20.6-41.9)	28.3 (24.9-42.9)	24.4 (20.0-67.0)
Intensive care, n (%)	0	1 (10%)	0
HFNC Oxygen, n (%)	0	7 (70%)	0
EQ5D VAS, median (min-max)	40 (0-87)	70 (20-80)	87 (30-100)
Recruitment period	2021-12 – 2022-11	2021-12 – 2022-06	2022-07 – 2022-12
Repeated blood sampling, n (%)	20 (53%)	6 (60%)	–
Months from acute infection to 1^st^ available sample, median (min-max)	15 (9-24)	16 (6-26)	10 (5-27)
Months from acute infection to 2^nd^ available sample, median (min-max)	24 (21-33)	24 (13-33)	–
Comorbidities, n (%)			
Psychiatric disease	2 (7%)	1 (11%)	4 (6%)
Allergy	0 (0%)	1 (11%)	4 (6%)
Pulmonary disease	3 (10%)	0 (0%)	6 (9%)
Vascular disease	0 (0%)	3 (33%)	12 (18%)
Ischemic heart disease	0 (0%)	1 (11%)	0 (0%)
Arrythmia	2 (7%)	0 (0%)	2 (3%)
Neurological disease	2 (7%)	3 (33%)	5 (7%)
Thyroid disease	4 (14%)	0 (0%)	2 (3%)
Diabetes mellitus	0 (0%)	1 (11%)	0 (0%)
Other endocrinological disease	0 (0%)	1 (11%)	0 (0%)
Haematological disease	0 (0%)	0 (0%)	1 (1%)
Gynaecological disease	2 (7%)	1 (11%)	2 (3%)
Urological disease	0 (0%)	0 (0%)	2 (3%)
Gastrointestinal disease	4 (14%)	2 (22%)	3 (4%)
Disease of the musculoskeletal system	3 (10%)	1 (11%)	3 (4%)
Ophthalmological disease	0 (0%)	0 (0%)	2 (3%)
Dermatological disease	2 (7%)	1 (11%)	0 (0%)
Malignancies	0 (0%)	0 (0%)	2 (3%)
Fibromyalgia	2 (7%)	2 (22%)	0 (0%)
POTS	1 (3%)	0 (0%)	0 (0%)
Symptoms, n (%)			
Fatigue	33 (87%)	7 (70%)	23 (29%)
Headache	30 (79%)	6 (60%)	12 (15%)
Dizziness	33 (87%)	7 (70%)	3 (4%)
Fever	13 (34%)	1 (10%)	1 (1%)
Cognitive symptoms	35 (92%)	8 (80%)	5 (6%)
Dyspnoea	34 (89%)	9 (90%)	8 (10%)
Chest pain	25 (66%)	2 (20%)	1 (1%)
Palpitations	30 (79%)	4 (40%)	4 (5%)
Pain	27 (73%)	5 (50%)	3 (4%)
Paraesthesia	24 (63%)	5 (50%)	2 (3%)
Vaccination status, n (%)			
Vaccinated before infection	2 (7%)	0 (0%)	32 (46%)
Vaccinated before sampling	12 (44%)	5 (63%)	30 (43%)
Vaccinated, date not specified	3 (11%)	2 (25%)	5 (7%)
Not vaccinated	10 (37%)	1 (13%)	3 (4%)

Comorbidities, vaccination status and symptoms are self-reported.

PASC, Post-Acute Sequelae of COVID-19; HFNC, High-Flow Nasal Cannula.

Demographic data are displayed in **Table 1**. In the patient group with a mild SARS-CoV-2 infection, median age was lower compared to the patients with a severe infection (p=0.008) and controls (p<0.0001). The most common PASC-related symptoms in both groups were cognitive impairment, dyspnoea, and fatigue. As a measure of health-related quality of life, the participants marked their perception of their current health status between 0–100 with the EQ-5D Visual Analogue Scale (VAS) where 0 represents the worst imaginable health and 100 represents the best imaginable health. PASC patients who had a severe acute COVID-19 disease had a median VAS of 70 (range 20-80), PASC patients who had a mild acute COVID-19 disease had a median VAS of 40 (range 0-87) and the controls had a median VAS of 87 (range 30-100). Despite the markedly lower median VAS score in the mild group, there was no significant difference between the patients with mild and severe acute COVID-19 disease, presumably due to the large inter-individual differences as well as the small sample size of the severe group.

### Activation of complement factors

**Figure 2:**
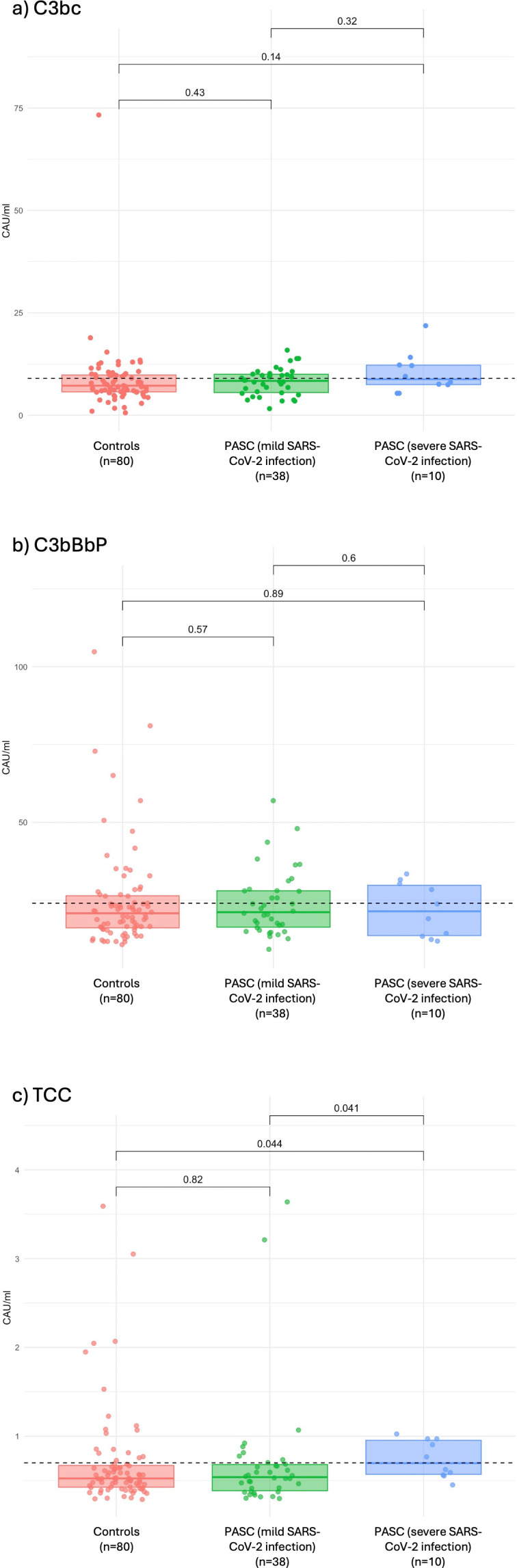
Results of complement activation markers **(a)** C3bc, **(b)** C3bBbP and **(c)** TCC. Complement activation markers are measured in CAU/mL. Results are demonstrated for the first available sample for PASC patients with a mild and severe acute infection and for control subjects. Differences between groups are tested with the Mann–Whitney U test. A p-value less than 0.05 is considered statistically significant. P-values were unadjusted for multiple comparisons. The dotted line represents the upper normal limit as suggested by Bergseth et al. (16).

Plasma samples were collected from both patients and control subjects at inclusion, and from patients again at follow-up visits, and were subsequently analysed for the complement activation products C3bc (common pathway), C3bBbP (alternative pathway) and TCC (terminal pathway). In the first available sample from each individual, no statistically significant differences in C3bc and C3bBbP levels were detected between the two groups of PASC patients and the control group (**Figure 2**). However, TCC was statistically significantly higher in the patients who had a severe SARS-CoV-2 infection compared to the patients who had a mild acute infection and controls (median 0.70 vs. 0.54 CAU/mL, p=0.041 and median 0.70 vs. 0.52 CAU/mL, p=0.044, respectively), indicating that terminal pathway activation could be associated with severe, but not mild, SARS-CoV-2 infection, but importantly not with PASC itself. C3bc levels in one of the control samples were below the limit of detection and excluded; all other values were within measurable range.

As a secondary analysis, we analysed the subgroup of patients with acute mild (n=20) and acute severe (n=6) SARS-CoV-2 infections who returned for resampling (**Supplementary Figure 1**). C3bc, C3bBbP and TCC values were not statistically significantly different between the two patient groups compared to controls in neither the first nor the second sample (**Supplementary Figure 1**). Although there was a statistically significant increase in the activation of C3bBbP between the second and first sample in the mild group, neither sample did significantly differ from controls, suggesting a possible individual variation over time not related to the presence of PASC.

To account for differences in the time from infection to sampling between controls and PASC patients, sensitivity analyses were performed. Log-transformed levels of the three complement factors were analysed using multivariable linear regression models fitted separately for each factor and adjusted for time since infection, age, sex and group (controls, mild initial COVID-19 and severe initial COVID-19). No covariate was significantly associated with any complement factor, and no differences were observed between controls and PASC patients (**Supplementary Table 2**).

In an additional sensitivity analysis, all PASC patients were analysed as a combined group. Results were unchanged, with no significant associations between any covariate—including PASC status—and any complement factor (**Supplementary Table 3**).

## Discussion

In this study of 48 well-characterized patients with PASC, we found no evidence of complement activation in plasma among those with an initially mild infection, despite severe PASC symptoms. Several studies have analysed changes in the complement system in patients with PASC, addressing complement activation, complement function and complement biomarkers (8–12, 17), summarized in a recent review (18). Baillie et al. studied a cohort of individuals with PASC and compared them to matched healthy convalescent individuals and detected activation of the classical (C1s-C1INH complex), alternative (Ba, iC3b) and terminal (C5a, TCC) pathways (8). Cervia-Hasler et al. employed a proteomics approach and found increased complement terminal pathway activation in patients with PASC compared to controls, both during the acute infection and at 6 months follow-up (9). This imbalance was marked by increased soluble C5bC6 complexes and decreased levels of C7-containing TCC (C5b-9) formations, suggesting increased membrane insertion of TCC (membrane attack complex), contributing to tissue damage in patients with PASC. However, these proteomics data were recently reanalysed in a non-peer-reviewed study by Farztdinov et al., who found no evidence of PASC-related complement activation after adjustment for age, body mass index, and sex imbalances (17).

Since no established PASC criteria exist in clinical practice, study design is a challenge. The lack of PASC biomarkers and diagnostic tools present further obstacles leading to notable heterogenic patient cohorts. The different results can likely be related to different methodological approaches, different study populations and varying follow-up times, although we raise concern about some methodological issues, such as the use of serum for the analysis of complement activation in the study by Cervia-Hasler (9). Complement activation markers are significantly higher in serum than in EDTA plasma; therefore, complement activation should be assessed only in plasma containing at least 10 mM EDTA and frozen immediately at −80 °C to limit pre-analytical *in vitro* activation (19, 20).

Our study has some major strengths in cohort design, which in some ways differ from the studies which showed complement activation in patients with PASC. Firstly, our patients were recruited from a specialized PASC clinic to which they required a referral from another health care provider, which suggests a selection of patients with more severe PASC symptoms, as indicated by the low reported EQ5D-VAS score. In some of the other studies, the patients were recruited from large cohorts of individuals with previously known SARS-CoV-2 infections, who acknowledged persistent symptoms although the severity of symptoms is not described (9, 12). Secondly, we analysed individuals with an initially mild infection separately from those with an initial severe infection. In some of the other studies these groups were analysed together, or the severity of the acute infection was not described (9, 11). Furthermore, the majority of the patients were hospitalized in some of the studies, suggesting a severe acute infection (9, 12). It is likely that patients with an initially severe and patients with an initially mild infection differ in the degree of persisting complement activation and therefore it is important to analyse these groups separately. Notably, we did identify a modest statistically significant elevation of TCC in the group having had an acute severe infection. However, the median TCC level in patients with severe SARS-CoV-2 infection (0.7 CAU/mL) was right at the upper limit of the normal reference range (<0.7 CAU/mL). The clinical relevance of this slight and isolated activation remains uncertain and the underlying mechanisms may be an unrelated variability as well as comorbid conditions or persistent immune activation. Thirdly, the time from infection to blood sampling was in median 16 months in our study, compared to as short as 3–6 months in some studies (9, 10, 12). It is possible that the complement system is temporarily affected following infection in PASC patients, but this effect may not persist even when symptoms remain. Lastly, we conducted targeted analyses instead of broad proteomics used in the majority of the other studies (9, 11, 12). Another strength of this study is that all included patients and control subjects had a documented acute infection with SARS-CoV-2 and underwent thorough interviews on symptom fluctuations. This evaluation was performed by two study physicians to ensure consistent interpretation of patient history.

We also acknowledge the limitations of the study, such as the diagnostic uncertainty inherently associated with a diagnosis based on self-reported symptoms as well as relatively small patient cohorts and control groups. For example, the subgroup of patients who initially had a severe infection with SARS-CoV-2 is small (n=10), leading to a potential type II error. Furthermore, there is no complete matching between patients and control subjects and therefore the variant of SARS-CoV-2 and the time between infection to blood sampling differ between the groups. Generally, individuals infected earlier in the pandemic showed a higher risk of developing PASC symptoms (21). Even though the PASC patients in our cohort were infected earlier in the pandemic than the control subjects we could not find a difference in complement activation, which strengthens the results despite the groups not being matched. Our aim was to show whether individuals with symptoms of PASC have an ongoing activation of the complement system. Although the PASC patients experienced a multitude of symptoms our results showed no increase in complement activation compared to controls or population reference value, therefore ongoing complement activation cannot explain their symptoms. However, we cannot exclude that they may have had complement activation earlier in the course of the disease. Complement activation has previously been shown to occur during the acute phase of the disease (22). Similarly, we cannot exclude localized or compartmental activation of the complement system. Another limitation of the study is that vaccination status was not included as a covariate in the analyses, which may influence markers of immune response.

In conclusion, we could not find convincing evidence of ongoing activation of the complement system in patients with PASC due to an acute mild SARS-CoV-2 infection, the most frequent clinical scenario preceding this condition (5). Thus, our data do not support the theory of complement activation as a key pathophysiological mechanism.

## Data Availability

Due to the inclusion of personal health details, the dataset cannot be openly shared, as mandated by the ethical review board. However, data will be made available upon reasonable request after publication and after confirming that ethical approval has been obtained. Requests to access the datasets should be directed to fredrik.kahn@med.lu.se.
