# Supplementary material for: Complement activation in patients with post-acute sequelae after SARS-CoV-2 infection
Source: Front Immunol. 2026 May 13;17:1779393. doi: 10.3389/fimmu.2026.1779393 (PMC13212466; doi:10.3389/fimmu.2026.1779393)
Supplement: Supplementary file 1 [file Image1.pdf]

Supplementary figure 1:

a) C3bc

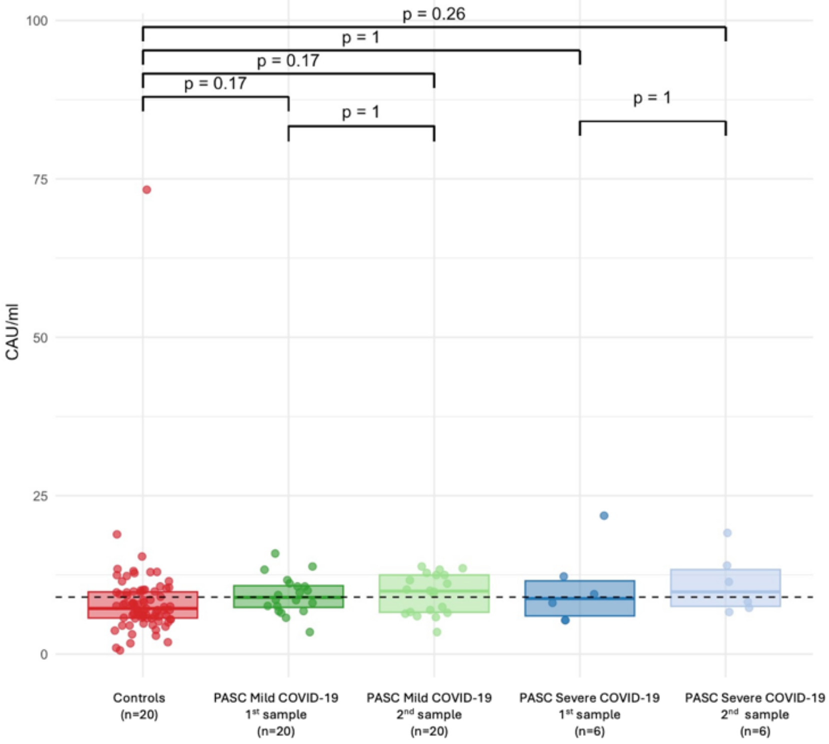

b) C3bBbP

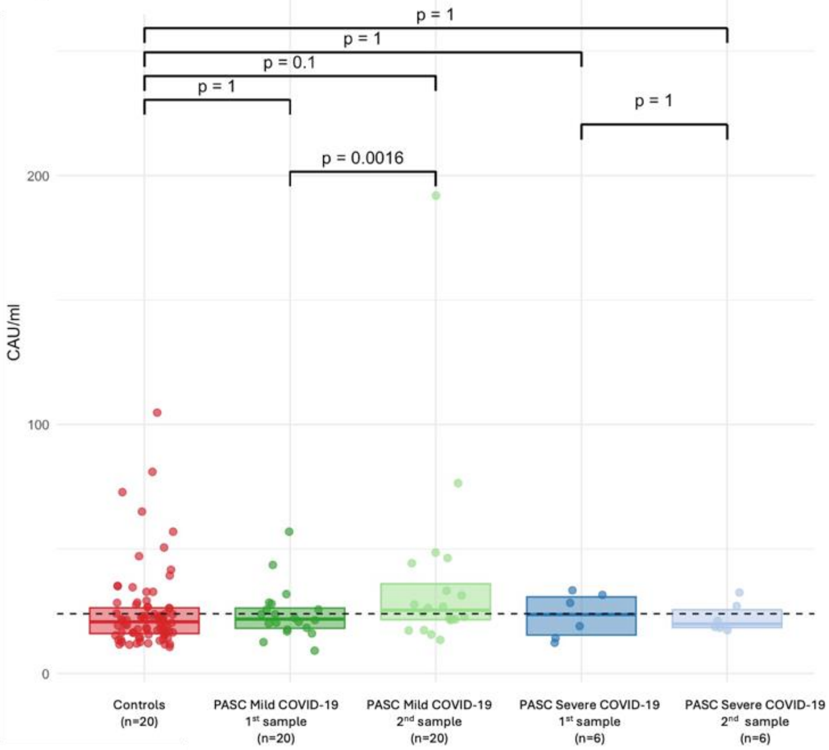

c) TCC

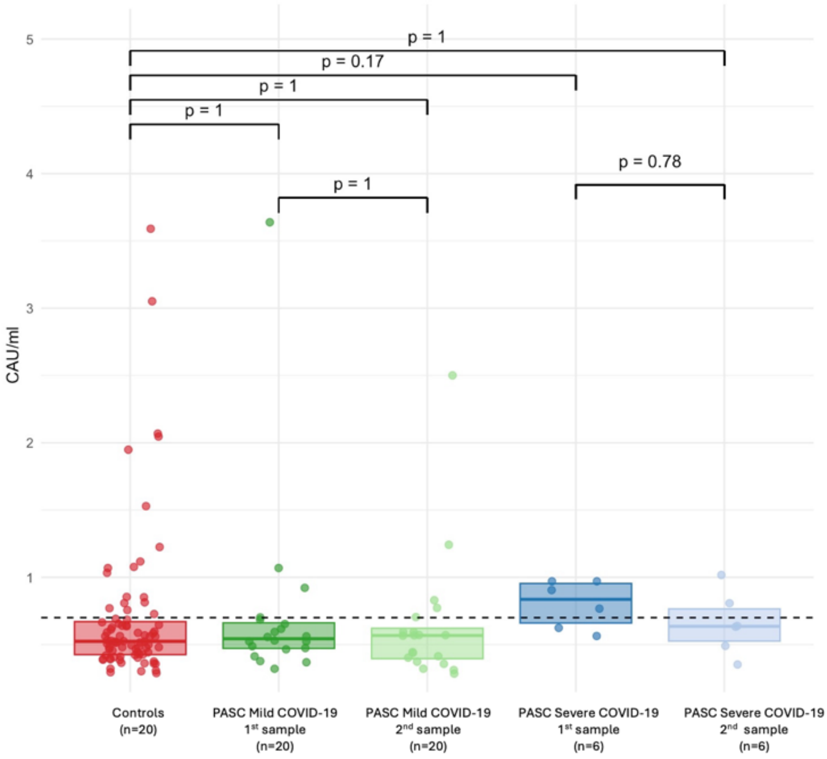

Secondary analysis of repeated measurement of complement activation markers a) C3bc, b) C3bBbP and c) TCC. Complement activation markers are measured in CAU/mL. Differences between group medians are tested with the Mann–Whitney U test and differences between paired measurements are tested with the Wilcoxon signed rank test. A p-value less than 0.05 is considered statistically significant. P-values are adjusted within every complement activation marker respectively, using the Holm-Bonferroni method to accommodate multiple comparisons. The dotted line represents the upper normal limit as suggested by Bergseth et al [16].
