# Supplementary material for: Complement activation in patients with post-acute sequelae after SARS-CoV-2 infection
Source: Front Immunol. 2026 May 13;17:1779393. doi: 10.3389/fimmu.2026.1779393 (PMC13212466; doi:10.3389/fimmu.2026.1779393)
Supplement: Supplementary file 2 [file Table1.docx]

**Supplementary table 1:**

|  | PASC (mild COVID-19)  n=38 | PASC (severe COVID-19)  n=10 | Controls  n=80 |
| --- | --- | --- | --- |
| Age, n (%) | 0 | 0 | 0 |
| Female sex, n (%) | 0 | 0 | 0 |
| BMI, n (%) | 9 (24%) | 2 (20%) | 10 (8%) |
| Self-reported comorbidities, n (%) | 9 (24%) | 1 (10%) | 13 (16%) |
| Months since acute infection to 1^st^ available sample, n (%) | 0 | 0 | 0 |
| Months since acute infection to 2^nd^ available sample, n (%) | 0 | 0 | - |
| Symptoms, n (%) | 0-1 (0-3%) | 0 | 0-1 (0-1%) |
| EQ5D VAS, n (%) | 9 (24%) | 1 (10%) | 13 (16%) |
| Intensive care, n (%) | 0 | 0 | 0 |
| HFNC Oxygen, n (%) | 0 | 0 | 0 |

Number and proportion of missing values from table 1.
