# Supplementary material for: Complement activation in patients with post-acute sequelae after SARS-CoV-2 infection
Source: Front Immunol. 2026 May 13;17:1779393. doi: 10.3389/fimmu.2026.1779393 (PMC13212466; doi:10.3389/fimmu.2026.1779393)
Supplement: Supplementary file 3 [file Table2.docx]

**Supplementary table 2:** **Multivariable linear regression models**

1. Regression coefficients for log-transformed C3bc

| **Variable** | **B** | **95% CI** | **β** | **t** | **p** |
| --- | --- | --- | --- | --- | --- |
| Time since infection (months) | 0.004 | [-0.014, 0.022] | 0.038 | 0.405 | 0.686 |
| Age | -0.002 | [-0.013, 0.009] | -0.039 | -0.396 | 0.693 |
| Female sex | -0.011 | [-0.238, 0.217] | -0.008 | -0.092 | 0.927 |
| PASC with mild COVID-19 | 0.033 | [-0.213, 0.279] | 0.026 | 0.264 | 0.792 |
| PASC with severe COVID-19 | 0.293 | [-0.098, 0.684] | 0.139 | 1.483 | 0.141 |

Note. R^2^adj = -0.018

1. Regression coefficients for log-transformed C3bBbP

| **Variable** | **B** | **95% CI** | **β** | **t** | **p** |
| --- | --- | --- | --- | --- | --- |
| Time since infection (months) | -0.011 | [-0.025, 0.003] | -0.142 | -1.527 | 0.129 |
| Age | -0.001 | [-0.010, 0.007] | -0.028 | -0.285 | 0.776 |
| Female sex | -0.014 | [-0.192, 0.164] | -0.014 | -0.153 | 0.879 |
| PASC with mild COVID-19 | 0.031 | [-0.162, 0.223] | 0.032 | 0.316 | 0.753 |
| PASC with severe COVID-19 | -0.026 | [-0.332, 0.280] | -0.016 | -0.167 | 0.867 |

Note. R^2^adj = -0.019

1. Regression coefficients for log-transformed TCC

| **Variable** | **B** | **95% CI** | **β** | **t** | **p** |
| --- | --- | --- | --- | --- | --- |
| Time since infection (months) | -0.013 | [-0.028, 0.003] | -0.151 | -1.645 | 0.102 |
| Age | 0.002 | [-0.008, 0.011] | 0.038 | 0.391 | 0.697 |
| Female sex | 0.128 | [-0.069, 0.325] | 0.115 | 1.283 | 0.202 |
| PASC with mild COVID-19 | 0.024 | [-0.190, 0.237] | 0.022 | 0.219 | 0.827 |
| PASC with severe COVID-19 | 0.253 | [-0.087, 0.592] | 0.136 | 1.474 | 0.143 |

Note. R^2^adj = 0.012
