# Supplementary material for: Complement activation in patients with post-acute sequelae after SARS-CoV-2 infection
Source: Front Immunol. 2026 May 13;17:1779393. doi: 10.3389/fimmu.2026.1779393 (PMC13212466; doi:10.3389/fimmu.2026.1779393)
Supplement: Supplementary file 4 [file Table3.docx]

**Supplementary table 3:** **Multivariable linear regression models with combined PASC group**

1. Regression coefficients for log-transformed C3bc

| **Variable** | **B** | **95% CI** | **β** | **t** | **p** |
| --- | --- | --- | --- | --- | --- |
| Time since infection (months) | 0.004 | [-0.014, 0.022] | 0.044 | 0.472 | 0.638 |
| Age | 0.000 | [-0.011, 0.010] | -0.002 | -0.020 | 0.984 |
| Female sex | -0.013 | [-0.241, 0.215] | -0.010 | -0.113 | 0.910 |
| PASC | 0.097 | [-0.126, 0.320] | 0.083 | 0.860 | 0.391 |

Note. R^2^adj = -0.021

1. Regression coefficients for log-transformed C3bBbP

| **Variable** | **B** | **95% CI** | **β** | **t** | **p** |
| --- | --- | --- | --- | --- | --- |
| Time since infection (months) | -0.011 | [-0.025, 0.003] | -0.144 | -1.553 | 0.123 |
| Age | -0.002 | [-0.010, 0.006] | -0.038 | -0.412 | 0.681 |
| Female sex | -0.013 | [-0.191, 0.164] | -0.013 | -0.148 | 0.883 |
| PASC | 0.017 | [-0.157, 0.190] | 0.018 | 0.192 | 0.848 |

Note. R^2^adj = -0.011

1. Regression coefficients for log-transformed TCC

| **Variable** | **B** | **95% CI** | **β** | **t** | **p** |
| --- | --- | --- | --- | --- | --- |
| Time since infection (months) | -0.012 | [-0.028, 0.003] | -0.145 | -1.577 | 0.117 |
| Age | 0.004 | [-0.005, 0.013] | 0.075 | 0.812 | 0.418 |
| Female sex | 0.126 | [-0.072, 0.323] | 0.113 | 1.259 | 0.210 |
| PASC | 0.080 | [-0.113, 0.273] | 0.077 | 0.819 | 0.414 |

Note. R^2^adj = 0.008
